# A127 PEMBROLIZUMAB-ASSOCIATED IMMUNE CHECKPOINT INHIBITOR GASTRITIS AND DUODENITIS: A CASE REPORT

**DOI:** 10.1093/jcag/gwae059.127

**Published:** 2025-02-10

**Authors:** J Zhen, S Canning, M Rushton

**Affiliations:** Department of Medicine, The Ottawa Hospital, Ottawa, ON, Canada; Division of Gastroenterology, Department of Medicine, The Ottawa Hosptal, Ottawa, ON, Canada; Division of Medical Oncology, Department of Medicine, The Ottawa Hospital, Ottawa, ON, Canada

## Abstract

**Background:**

Advancements in immunotherapy have revolutionized the treatment of cancer. However, innate to their mechanism of action, these therapies are associated with various immune-related adverse effects which present challenges to their use. While immune checkpoint inhibitor-associated colitis is the most common gastrointestinal toxicity, upper gastrointestinal involvement, such as esophagitis, gastritis, and duodenitis remains considerably rare.

**Aims:**

The aim of this case report is to describe a severe case of immune checkpoint inhibitor-associated gastritis/duodenitis and highlight some of the challenges linked with its diagnosis and management.

**Methods:**

We performed a retrospective chart review of the described patient and conducted a review of the relevant literature.

**Results:**

A 65-year-old female was diagnosed with triple-negative, invasive ductal carcinoma and had been undergoing adjuvant treatment with pembrolizumab. Ten months after initiating therapy, she presented with epigastric pain, nausea/vomiting, and poor appetite. She subsequently underwent an esophagogastroduodenoscopy (EGD) which revealed a firm and friable stomach with thickened mucosa and significant edema of the duodenal cap. An initial review of the biopsies taken during upper endoscopy demonstrated non-specific gastric and duodenal inflammation without a definitive diagnosis. However, a subsequent review at consensus rounds suggested severe immune checkpoint inhibitor-associated gastritis/duodenitis and she was started on corticosteroid therapy. While her symptoms were refractory to high-dose intravenous methylprednisolone, she showed marked improvement after two infusions of infliximab (5mg/kg and 10mg/kg, respectively).

**Conclusions:**

This case highlights the importance of increasing awareness of immune checkpoint inhibitor-associated gastritis and duodenitis, involving a multidisciplinary team to help facilitate its diagnosis, and emphasizes the role of repeated doses of biologic therapy in steroid-refractory cases.

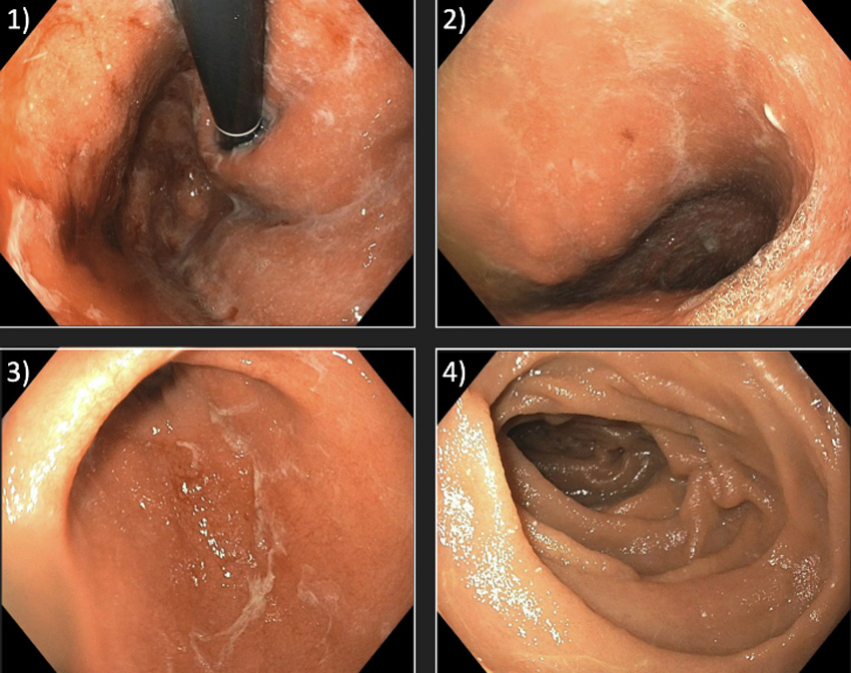

**Figure 1.** Endoscopic findings of immune checkpoint inhibitor-associated gastritis (1) Diffuse edema and friability in the gastric fundus. (2) Diffusely edematous gastric body with loss of the typical gastric folds. (3) Mild edema along the gastric antrum. (4) Normal appearing duodenal mucosa in the second portion of the duodenum.

**Funding Agencies:**

None

